# Radiosensitivity in patients affected by ARPC1B deficiency: a new disease trait?

**DOI:** 10.3389/fimmu.2022.919237

**Published:** 2022-07-29

**Authors:** Maria Chiriaco, Giorgiana Madalina Ursu, Donato Amodio, Nicola Cotugno, Stefano Volpi, Francesco Berardinelli, Simone Pizzi, Cristina Cifaldi, Matteo Zoccolillo, Ignazia Prigione, Silvia Di Cesare, Carmela Giancotta, Elisa Anastasio, Beatrice Rivalta, Lucia Pacillo, Paola Zangari, Alessandro G. Fiocchi, Andrea Diociaiuti, Alessandro Bruselles, Francesca Pantaleoni, Andrea Ciolfi, Valentina D’Oria, Giuseppe Palumbo, Marco Gattorno, Maya El Hachem, Jean-Pierre de Villartay, Andrea Finocchi, Paolo Palma, Paolo Rossi, Marco Tartaglia, Alessandro Aiuti, Antonio Antoccia, Gigliola Di Matteo, Caterina Cancrini

**Affiliations:** ^1^ Department of Systems Medicine, University of Rome Tor Vergata, Rome, Italy; ^2^ Academic department of Pediatrics, Research Unit of Primary Immunodeficiencies, Bambino Gesù Children’s Hospital, Scientific Institute for Research and Healthcare (IRCCS), Rome, Italy; ^3^ Academic Department of Pediatrics, Research Unit of Clinical Immunology and Vaccinology, Bambino Gesù Children’s Hospital, Rome, Italy; ^4^ Center for Autoinflammatory Diseases and Immunodeficiencies, Scientific Institute for Research and Healthcare (IRCCS) Istituto Giannina Gaslini and University of Genoa, Genoa, Italy; ^5^ Laboratory of Neurodevelopment, Neurogenetics and Molecular Neurobiology, Scientific Institute for Research and Healthcare (IRCCS) Santa Lucia Foundation, Rome, Italy; ^6^ Department of Science, Roma Tre University, Rome, Italy; ^7^ Genetics and Rare Diseases Research Division, Bambino Gesù Children’s Hospital, Scientific Institute for Research and Healthcare (IRCCS), Rome, Italy; ^8^ San Raffaele Telethon Institute for Gene Therapy, Scientific Institute for Research and Healthcare (IRCCS), San Raffaele Scientific Institute, Milan, Italy; ^9^ Department of Medical and Surgical Sciences, Pediatrics Unit, University “Magna Graecia”, Catanzaro, Italy; ^10^ Pediatric Allergology Unit, Bambino Gesù Children’s Hospital, Scientific Institute for Research and Healthcare (IRCCS), Rome, Italy; ^11^ Dermatology Unit, Bambino Gesù Children’s Hospital, Scientific Institute for Research and Healthcare (IRCCS), Rome, Italy; ^12^ Department of Oncology and Molecular Medicine, Istituto Superiore di Sanità, Rome, Italy; ^13^ Research Laboratories, Bambino Gesù Children’s Hospital, Scientific Institute for Research and Healthcare (IRCCS), Rome, Italy; ^14^ Department of Haematology, Bambino Gesù Children’s Hospital, Scientific Institute for Research and Healthcare (IRCCS), Rome, Italy; ^15^ Université de Paris, Imagine Institute, Laboratory “Genome Dynamics in the Immune System”, INSERM UMR 1163, F-75015, Paris, France; ^16^ Pediatric Immunohematology, San Raffaele Scientific Institute, Milan, Italy; ^17^ Vita Salute San Raffaele University, Milan, Italy

**Keywords:** ARPC1B, combined immunodeficiency, immune dysregulation, radiosensitivity, DNA damage response (DDR)

## Abstract

Actin-related protein 2/3 complex subunit 1B (ARPC1B) deficiency is a recently described inborn error of immunity (IEI) presenting with combined immunodeficiency and characterized by recurrent infections and thrombocytopenia. Manifestations of immune dysregulation, including colitis, vasculitis, and severe dermatitis, associated with eosinophilia, hyper-IgA, and hyper-IgE are also described in ARPC1B-deficient patients. To date, hematopoietic stem cell transplantation seems to be the only curative option for patients. ARPC1B is part of the actin-related protein 2/3 complex (Arp2/3) and cooperates with the Wiskott–Aldrich syndrome protein (WASp) in the regulation of the actin cytoskeleton remodeling and in driving double-strand break clustering for homology-directed repair. In this study, we aimed to investigate radiosensitivity (RS) in ARPC1B-deficient patients to assess whether it can be considered an additional disease trait. First, we performed trio-based next-generation-sequencing studies to obtain the ARPC1B molecular diagnosis in our index case characterized by increased RS, and then we confirmed, using three different methods, an increment of radiosensitivity in all enrolled ARPC1B-deficient patients. In particular, higher levels of chromatid-type aberrations and γH2AX foci, with an increased number of cells arrested in the G2/M-phase of the cell cycle, were found in patients’ cells after ionizing radiation exposition and radiomimetic bleomycin treatment. Overall, our data suggest increased radiosensitivity as an additional trait in ARPC1B deficiency and support the necessity to investigate this feature in ARPC1B patients as well as in other IEI with cytoskeleton defects to address specific clinical follow-up and optimize therapeutic interventions.

## Highlights

- Patient with a homozygous deletion c.212_226del in *ARPC1B* gene presenting with combined immunodeficiency, recurrent infections, thrombocytopenia, immune dysregulation, and increased radiosensitivity.
- Radiosensitivity is a new trait of ARPC1B defect.
- The arrest of damaged cells in the G2/M-phase is suggestive of a defective Arp2/3-ARPC1B complex that is unable to drive DNA double-strand breaks (DSBs) clustering for homology-directed repair (HDR) but also of an impaired Aurora-A activation.
- To study radiosensitivity in ARPC1B-deficient patients as well as in other IEI with cytoskeleton defects is needed to optimize therapeutic interventions.

## Introduction

Actin-related protein 2/3 complex subunit 1B (ARPC1B) deficiency is a recently described autosomal recessive disease characterized by combined immunodeficiency (CID), thrombocytopenia, and immune dysregulation having eczema, allergy, autoimmunity, and inflammatory diseases, in addition to an increased risk of severe infections as common features (1–6). ARPC1B is mostly expressed in hematopoietic cells. It is one of the two ARPC1 isoforms belonging to the actin-related protein 2/3 (Arp2/3) complex, which is essential for actin nucleation and polymerization in different critical cellular processes (e.g., phagocytosis, vesicular trafficking, and lamellipodia extension) and, as a centrosomal protein, for proper chromosome segregation during mitosis (7–9). After binding to the activated Wiskott–Aldrich syndrome protein (WASp), ARPC1B supports the assembly and stability of new actin filaments. Moreover, the Arp2/3 complex plays an essential role in DNA damage response (DDR) by promoting chromatin dynamics, driving double-strand break (DSB) clustering and repair by homology-directed repair (HDR) (7). Likewise, WASp plays a crucial role in the cell-protective and cell-repair mechanisms not only delimited in the nucleus but also involving the Golgi-dispersal response (GDR) in the cytoplasm (10). Although defects in actin polymerization explain several anomalies in chromosomal mobility and immune dysregulation, many aspects of the ARPC1B immunodeficiency are still poorly understood, and its role in DNA repair was never reported.

In the present study, starting from the characterization of a severe CID patient affected by ARPC1B deficiency presenting with an increased radiosensitivity (RS), we further investigated RS in other ARPC1B patients to assess whether it could be considered a trait of the disease.

## Methods (see supplementary data for further details)

### Patients and ethics statements

The case index patient (PtII-1) was enrolled, monitored, and treated during the follow-up at the Children’s Hospital Bambino Gesù (Rome). In addition, three previously molecularly and clinically defined ARPC1B patients (4, 5) were enrolled in the study to investigate the RS trait, other than WAS and AT patients who were analyzed as control subjects characterized by altered/increased radiosensitivity.

All procedures performed in the study were in accordance with the ethical standards of the institutional research committee and with the 1964 Helsinki declaration and its later amendments or comparable ethical standards. Blood samples, EBVB cell lines, and fibroblast cells were obtained from all enrolled subjects after obtaining informed consent following standard ethical procedures with approval of the Children’s Hospital Bambino Gesù Ethical Committee (1702_OPBG_2018), the Institutional Ethical Committee of San Raffaele Hospital (TIGET06, TIGET09), and Gaslini Hospital.

### Molecular investigations

External companies performed whole-exome sequencing (WES) and whole-genome sequencing (WGS) (https://www.genewiz.com/en-GB/Public/Services/Next-Generation-Sequencing/). WES and WGS statistics and data output are reported in **Supplementary Table S2**. WGS variants within coding regions are reported in **Supplementary Table S3**. Sanger sequencing on gDNA isolated from total PBMCs was performed to confirm the presence of mutations in *ARPC1B* (NCBI NM_005720.4) and *SLC6A19* (NCBI NM_001003841.3) genes (ABI PRISM 3130, Applied Biosystems, Foster City, CA).

### Radiosensitive assays

#### Irradiation

Epstein–Barr virus B (EBVB) cell lines were exposed at room temperature to 30 cGy X-rays (Gilardoni MGL 300/6D at 250 kV, 6 mA). After 3 h of incubation with 5 × 10^−6^ M colchicine, cells were harvested, and chromosome preparations were obtained using conventional methods. Hundred Giemsa-stained metaphases were scored for each experimental point in repeated independent experiments. For the scoring of γH2AX foci, fibroblasts were exposed to 1 Gy, fixed at 30 min, 2 h, 4 h, and 24 h, and the quantitative analysis of γH2AX was performed by counting by eye foci in at least 50 nuclei after counterstaining with DAPI (Zeiss Axiophot).

#### Bleomycin assay

EBVB cells were treated with chemical radiomimetic-induced DNA damage bleomycin (BLM) (9 µM, 1 h/37°C) and analyzed by FACS or IF for γH2AX at different time points of incubation (2, 4, 6, 8, 10, and 24 h) depending on the type of experiments. DNA damage repair, cell cycle phases, and cell viability were investigated. Details relative to FACS analysis and immunofluorescence/confocal laser microscopy protocols are reported in the Supplementary section.

## Results

### Characterization of the ARPC1B index case

A 12-year-old girl (PtII-1, herein referred to as the index case) born from consanguineous parents (**Supplementary Figure S1A**) was admitted at the age of 2 months for hematemesis, severe hemorrhagic diarrhea, perianal and oral ulcers, persistent eosinophilia, failure to thrive, and severe atopic dermatitis refractory to treatment. Her clinical course was complicated by *S. aureus* sepsis with an abscess in the site of central venous catheter (CVC) insertion. The family history resulted positive for allergy and psoriasis. During the follow-up period, we observed marked improvement in diarrhea and ulcerations, but the persistence of recurrent infections, mostly otitis (*P. aeruginosa*, *E. coli*, *P. mirabilis*) and severe psoriatic dermatitis with lymph and monocytic infiltrates (**Supplementary Figure S1B**). All the information related to the therapeutic treatment in the follow-up period was detailed and reported in the Supplementary section. Immune evaluation (**Supplementary Table S1A**) showed progressively increasing levels of IgA and total and antigen-specific IgE with the presence of ASMA, ANCA, anti-PLTs, and anticardiolipin autoantibodies (**Supplementary Table S1B**) together with mild thrombocytopenia. Monthly IVIG substitution and specific antibiotic treatment and prophylaxis were started. Although she had normal values of lymphocytes with polyclonal TCR repertoire and normal T-cell proliferation upon *in vitro* stimulations (PHA/OKT3/Candid/Tetanus toxoid), an increased memory T cell subset was confirmed over time, and a low memory B-cell subset was found. Additionally, progressive lymphopenia with an increment of CD21low memory cells was observed. Regulatory T cells (CD4^+^CD25^hi^CD127^low^Foxp3^+^) were also increased, showing mainly memory phenotype and reduced Helios (IKZF2) co-expression (**Supplementary Figure S2**) consistent with reduced suppressive activity (5). During the clinical follow-up CD4^+^CD45RO^+^CXCR5^+^ follicular helper T-cell (T_FH_) frequencies varied accordingly to the therapy received (**Supplementary Table S1A**). Additionally, T-cell proliferation.

Trio-based WES (**Supplementary Table S2**) was performed at 3 and 8 years of age, revealing a homozygous mutation (c.1606G>A; p.Val536Met) in the *SLC6A19* gene, encoding the amino acid transporter B^0^AT1. Mutations in this gene lead to Hartnup disorder (OMIM 234500) (11) (**Supplementary Figure S3**), a metabolic disease with variable clinical presentation, including sun-exposed pellagra-like rash. Increased urinary tryptophan levels confirmed the diagnosis, and she started a diet implemented with niacin with only a partial and transient improvement of dermatitis. At the age of 10, she had a subcutaneous administration of dupilumab (300 mg every 4 weeks) with significant improvement in both skin rash and itchiness. The progressive and severe clinical picture suggestive of IEI with immune dysregulation strongly pointed to the presence of an additional genetic defect/s as the underlying cause of the condition. A new analysis performed by trio-based WGS revealed (**Supplementary Table S3**) a homozygous in-frame 15-bp deletion, c.212_226del (p.Gly71_Asn75del) in the *ARPC1B* gene (**Supplementary Figure S3**). The variant had not been identified by two previous WES analyses, probably due to the inadequate coverage and poor quality of data. The ARPC1B protein was found absent in T, B, and NK cells and monocytes (by FACS) and in EBVB cells and T-blasts (by Western blot) (**Supplementary Figures S4A**, **B**). Moreover, accordingly to other ARPC1B patients (4, 5), PtII-1 patient’s T-blasts were less able to migrate in response to SDF1-a (**Supplementary Figure S4C**), confirming a defective cytoskeleton remodeling capacity and a loss-of-function (LOF) of ARPC1B protein.

### Increased ionizing radiation sensitivity in ARPC1B-deficient patients

In the frame of a study exploring RS in patients affected by CID, immune dysregulation, and/or severe dermatitis, before the identification of the *SLC6A19* and *ARPC1B* gene mutations, we investigated DSB repair in irradiated fibroblasts from our PtII-1 index case, revealing an increased RS assessed by a higher fraction of γH2AX foci (**Supplementary Figure S5**). Moreover, based on prior observations showing a defect in the TCRα repertoire in Ataxia Telangiectasia- (AT) and Nijmegen Breakage Syndrome- (NBS) patients, we quantified in PtII-1 and her parents (carriers of both identified mutations), the TCR-Vα7.2–expressing T-lymphocytes (FACS) and performed PROMIDISα analysis (12). We found a strong reduction of the T Vα7.2^+^/CD161^-^ subset associated with a decrease of CD45RA^+^/CD197^+^/CD4^+^-naïve T-lymphocytes (**Supplementary Figure S6A**). Moreover, PtII-1 harbored a PROMIDISα signature that clustered between those of patients with V(D)J/DNA repair deficiencies and AT deficiencies (AT49<Pt<AT8) (**Supplementary Figure S6B** *panel down*), while both parents’ signatures clustered with those of healthy donors (HDs) (C7<parents< C23) (**Supplementary Figure S6B** *panel down*).

**Figure 1:**
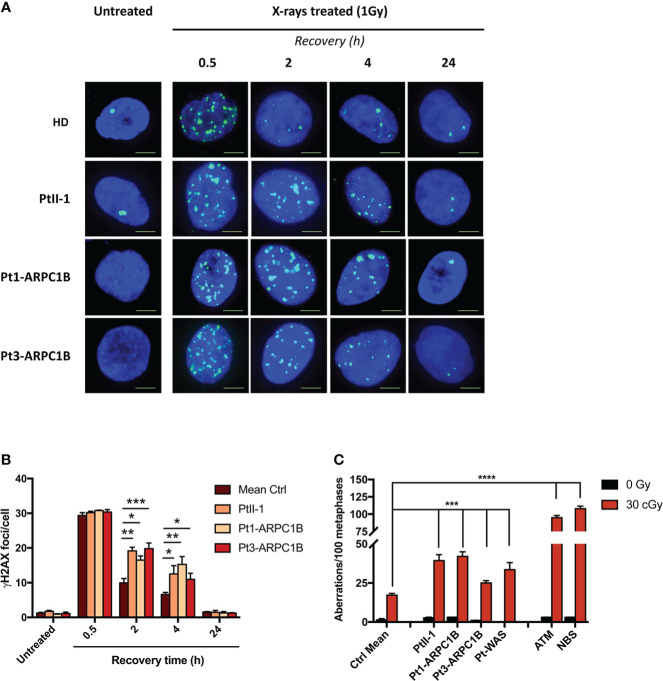
DNA DSB repair kinetics in healthy donors (HDs), PtII-1, Pt1-ARPC1B, and Pt3-ARPC1B. **(A)** Representative images of γH2AX staining in primary fibroblasts from HDs and patients irradiated with 1Gy X-rays and fixed after 0.5, 2, 4, and 24 h. **(B)** Histograms showing the number of γH2AX foci/cells in fibroblasts reported as mean ± SEM (*n* = 3; HDs = 3) at different time points after irradiation. **(C)** Frequency (mean ± SEM; *n* = 3; HDs = 6) of chromatid-type aberrations on irradiated EBVB cells in the G2-cell cycle phase. Statistical analysis was performed with a two-way ANOVA test and Tukey posttest. ^*^ *p* < 0.05; ^**^ *p* < 0.01; ^***^ *p* < 0.001; ^****^ *p* < 0.0001.

Extending the DSB repair analysis to irradiated fibroblasts from two other previously described ARPC1B patients (4, 5), we disclosed a high number of γH2AX foci (**Figures 1A**, **B**). In particular, the increment of γH2AX foci was statistically significant (^*^0.05 < *p*-value < ^***^0.001) at 2 and 4 h after treatment in all ARPC1B patients compared to HDs.

Furthermore, as shown in **Figure 1C**, the frequency of chromatid-type aberrations in EBVB irradiated in the G2-phase of the cell cycle was higher in ARPC1B patients, including PtII-1, compared to HDs, although at a lower level than AT and NBS patients’ derived cells (^***^ *p* < 0.001 vs. ^****^ *p* < 0.0001), two conditions highly sensitive to ionizing radiation (IR) (13). In addition, in line with a recent report (10), WAS patients’ cells (Pt1-WAS) showed increased sensitivity to IR (^***^ *p* < 0.001). The mutations found in ARPC1B and WAS patients are reported in **Supplementary Table S4**. These findings revealed an increased RS in all tested ARPC1B patients although with a lower level than those observed in AT and NBS syndromes.

### Increased bleomycin sensitivity at different stages of the cell cycle in ARPC1B-deficient patients

**Figure 2:**
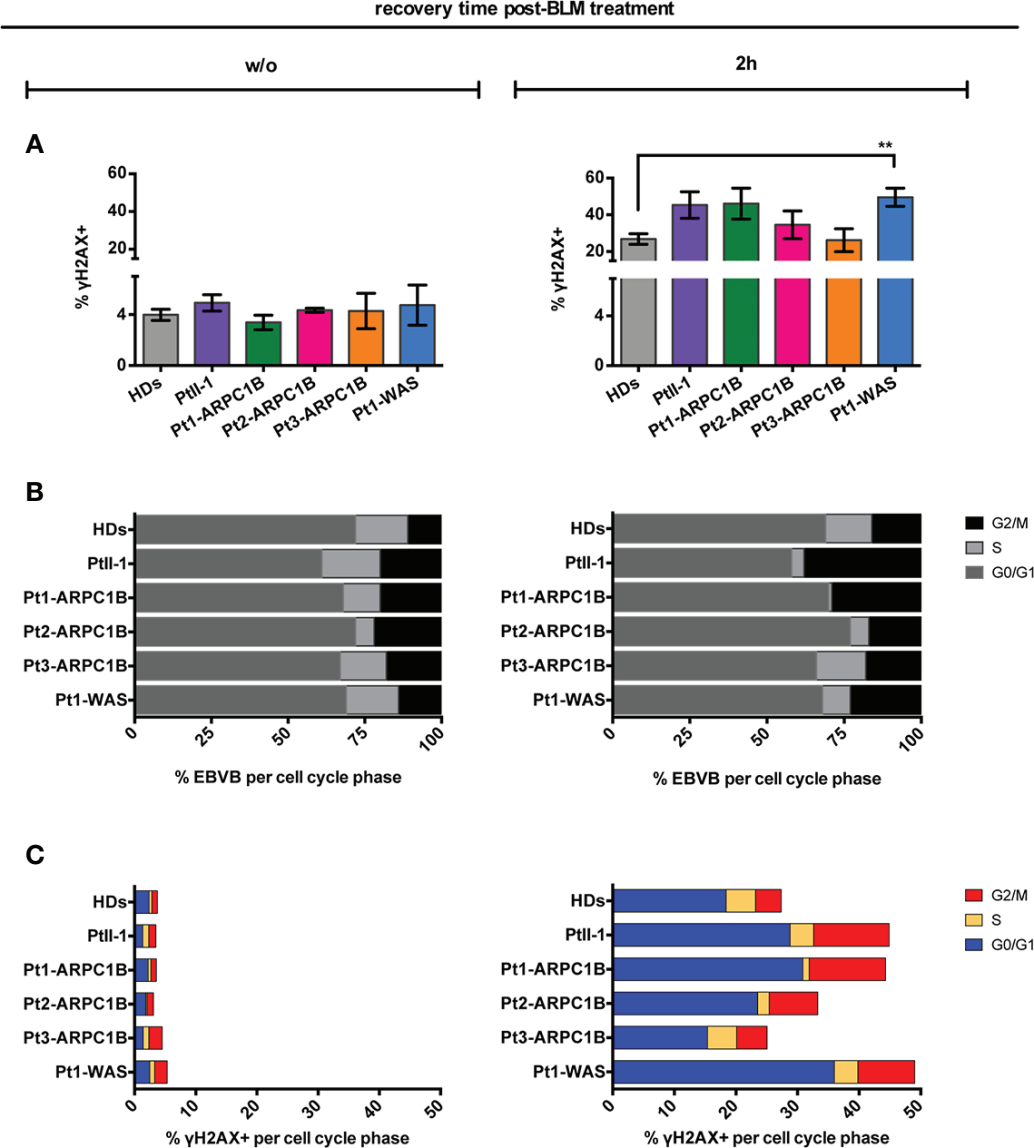
Radiosensitivity evaluation in ARPC1B-deficient cells after 2 h of bleomycin treatment. **(A)** FACS analysis of γH2AX expression in EBVB cells from HDs, Pt-II1, Pt1-3 ARPC1B, and Pt-WAS with or w/o BLM treatment (9 µM/1 h). Column bar graphs show the mean ± SEM (*n* > 3; HDs = 15). **(B)** Distribution of cells into the cell cycle phases before and after 2 h of BLM treatment (FACS). Each bar represents the mean of cells determined in at least three independent experiments. **(C)** Quantification of the γH2AX^+^ EBVB cells in each cell cycle phase (FACS). The percentage of cells in G0/G1, S, and G2/M cell cycle phases (γH2AX^+^ with respect to singlet cells) was represented as mean ± SEM (*n* > 3). Statistical significance was calculated with one-way ANOVA and Bonferroni’s posttest: ^**^ *p* < 0.01.

To further confirm our results, RS was explored in ARPC1B and WAS patients’ EBVB cells treated with the radiomimetic DNA-cleaving agent bleomycin (BLM) by assessing γH2AX expression through FACS and immunofluorescence assay (IF). The obtained results showed an increment of the γH2AX level in almost all ARPC1B-deficient patients’ cells following 2 h of treatment, which was more evident in PtII-1, Pt1-ARPC1B, and Pt1-WAS (**Figure 2A**). We also investigated cells obtained from PtII-1’s parents (carriers of mutations) that are in comparable to HDs and not radiosensitive (data not shown).

**Figure 3:**
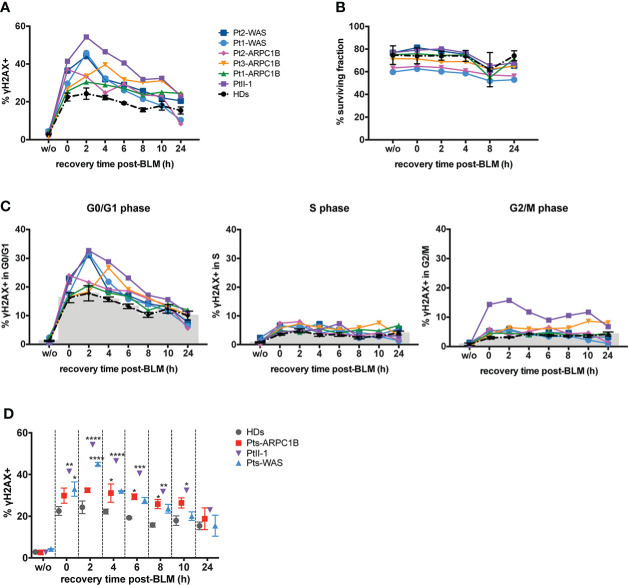
Repair-time kinetics of the cell survival fraction after BLM treatment. Kinetic experiment on untreated or BLM-treated EBVB cells from PtII-1, Pt1-3 ARPC1B patients, two WAS patients, and four HDs, investigated during 24 h of recovery time culture. **(A)** The graph shows the percentage of γH2AX^+^ EBVB cells. **(B)** Cell survival fractions were reported as the percentage of 7AAD^−^ EBVB cells. **(C)** Amount of γH2AX^+^ EBVB cells in each cell cycle phase: G0/G1 (left panel), S (middle panel), and G2/M (right panel). Black line indicates the mean ± SEM of the four HDs. **(D)** Percentage of γH2AX^+^ EBVB cells were reported as mean ± SEM calculated for each disease group (three ARPC1B patients and two WAS patients) and PtII-1 against HD group. Statistical significance was calculated with two-way ANOVA and Bonferroni’s posttest: ^*^ *p* < 0.05, ^**^ *p* < 0.01, ^***^ *p* < 0.001, and ^****^ *p* < 0.0001.

The characterization of cell cycle phases in patients’ EBVB cells revealed their different distribution during the cell cycle, with an expansion of the S- and G2/M-phases for all patients’ untreated cells and PtII-1, Pt1-ARPC1B, and Pt1-WAS after BLM treatment (**Figure 2B**). Notably, combining this evidence with the analysis of γH2AX expression in cell cycle phases, we highlighted an increased number of cells arrested in G2/M that expressed high levels of γH2AX in all patients’ BLM-treated cells, ranging up to threefold compared to HDs, with the exception of Pt3-ARPC1B (**Figure 2C**). To investigate the inter/intrapatient variability in terms of cell viability and γH2AX accumulation, we then performed kinetic experiments on EBVB cells from patients and HDs for 24 h of culture after BLM treatment (**Figure 3**). As reported in **Figure 3A**, all investigated patients showed a higher γH2AX level at almost all time points, albeit with different rates with respect to HDs. Interestingly, the analysis of surviving fractions (7AAD-negative cells) revealed a comparable viability profile in all samples, including HDs, although with a variable percentage of survival that was particularly reduced in some ARPC1B and WAS patients (**Figure 3B**). Of note, the cell cycle progression analysis confirmed an expansion of γH2AX^+^ cells upon BLM treatment, particularly evident in the G2/M-phase in PtII-1 (at all time points) and Pt3-ARPC1B1 cells (after 8 h) (**Figure 3C**). The comparison of ARPC1B and WAS patients with HD groups disclosed a statistically significant higher frequency of γH2AX during the first 8 h, although with slight differences between patients’ groups (**Figure 3D**), whereas PtII-1 showed the largest increase in RS over time compared to other patients (ARPC1B, WAS) and HDs.

**Figure 4:**
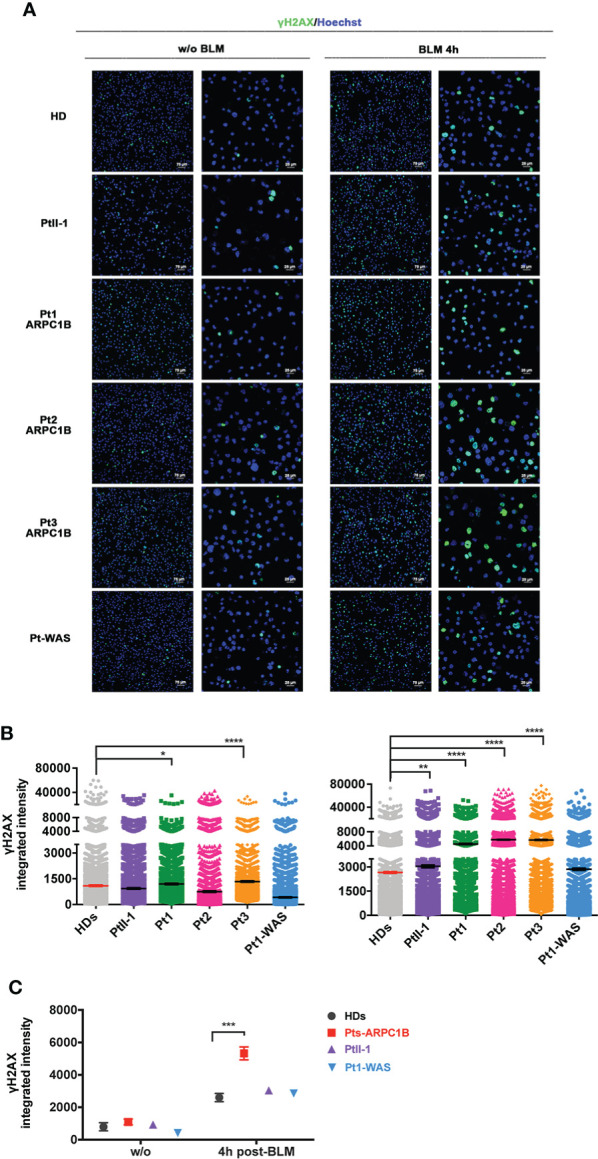
γH2AX expression in ARPC1B-deficient EBVB BLM-treated cells by immunofluorescence. **(A)** Representative images of γH2AX (green) and nuclei counterstaining (blue) investigated on cells from PtII-1, Pt1-3 ARPC1B, Pt1-WAS, and HD before and after 4 h of BLM treatment (9 µM/1 h). Each left column shows ×20 magnification, and each right column shows ×60 magnification. Bar scale represented 75 µm (×20) and 25 µm (×60). **(B)** Histograms show the γH2AX-integrated intensity evaluated in the patient’s EBVB cells compared to HDs (*n* = 4; HDs = 4). Each point represents the integrated intensity of a single cell. The line indicates the mean ± SEM (*n* > 2). **(C)** The graph shows a between-group statistical comparison of the γH2AX-integrated intensity detected in PtII-1, ARPC1B-, and WAS-disease group of patients against the HD group. The statistical analyses were performed with two-way ANOVA and Bonferroni’s posttest: ^*^ *p* < 0.05, ^**^ *p* < 0.01, ^***^ *p* < 0.001, and ^****^ *p* < 0.0001.

Finally, immunofluorescence analysis revealed a higher γH2AX protein expression in EBVB cells derived from ARPC1B- and WAS-deficient patients after 4 h of BLM treatment (**Figures 4A**, **B**). The γH2AX increment was statistically significant for all ARPC1B patients compared with HDs (^**^0.01 < *p*-value < ^****^0.0001) (**Figure 4C**). Interestingly, Pt3-ARPC1B, which displayed a borderline increased expression of γH2AX after 2 h of treatment by FACS, showed a higher increment after 4 h by both FACS and IF analysis. Moreover, cell radiosensitivity was also corroborated in Pt3’s derived fibroblasts along with an altered F-actin expression with abnormal branch morphology, suggesting a defective cytoskeleton organization (**Supplementary Figure S7**).

All this evidence seems to suggest a diverse time-dependent accumulation and clearance of γH2AX foci among the different ARPC1B and WAS patients.

## Discussion

The present study started with the investigation of a CID patient presenting with recurrent infections, thrombocytopenia, and immune dysregulation. Additionally, the patient showed increased memory T cells, low memory B cells, and increased Treg frequency with low Helios expression, other than an unbalanced Th2 polarization with increased IgE reactivity. Surprisingly, the patient’s cells showed an increased IR-induced RS.

Preliminary molecular studies revealed a homozygous mutation in the *SLC6A19* gene (11). Since the identified mutation did not explain the clinical and immunological phenotype and no evidence of RS has been described in Hartnup disease, we performed advanced molecular investigations. Following two inconclusive WES approaches, only a WGS allowed the identification of a second homozygous deletion in the *ARPC1B* gene, highlighting the importance of re-evaluating molecular and functional investigations of unresolved cases strongly suggestive of IEI on a periodic basis.

Thus, our patient represents the first case of ARPC1B deficiency in which an increased radiosensitivity was identified. Moreover, in contrast with other reported cases of ARPC1B patients (1–6), a marked increase of multiple autoantibodies and an increased percentage of CD21low B cells were detected since the first months of life. In addition, such immune perturbation was associated to severe autoimmunity at the gastrointestinal level in our patients, with symptoms mimicking very early inflammatory bowel disease. Indeed, an altered B-cell tolerance resulting from defective BCR stimulation due to an altered actin polymerization has recently been described (14).

In light of these preliminary findings, the purpose of this study was to investigate RS in other ARPC1B patients to evaluate if this trait can be considered a common hallmark. Indeed, although some IEI show higher RS (15, 16) and other ones show actin cytoskeletal defects (17–20), these two features were not considered together previously.

We confirmed an increased RS in ARPC1B-deficient patients using different methods, including the evaluation of chromatid aberrations and the γH2AX accumulation (21–23) in irradiated EBVB and fibroblast cells, respectively, or in bleomycin-treated EBVB cells by FACS and IF assays (22–24). Accordingly, in our index patient, we also found a decrement of TCR-Vα7.2 expressing T-lymphocytes and an altered PROMIDISα signature that could partially explain the immune dysregulated manifestations (12).

The use of phosphorylated γH2AX as a biomarker for RS has been supported by evidence showing that it is one of the earliest events during the DNA damage response and that its activation correlates with the rate of DSB re-joining (a sensitive marker of DSB repair) (21, 22). In light of this, an increment of γH2AX in the G2/M-phase after DNA damage induction could be explained by a defective HDR mechanism mostly in ARPC1B patients compared to the other WAS and AT patients. As reported by Schrank et al. (7), the arrest of damaged cells could be caused by a defective Arp2/3 complex in driving DNA DSB clustering for HDR repair during the G2-phase of the cell cycle. Differently, WASp has been demonstrated not only to specifically activate Arp2/3 at DSB sites undergoing HDR but also to exert its specific activity in DSB repair independently of the cell cycle stage, as ATM predominately does in the nonhomologous end-joining (NHEJ) mechanism repair (7). Although the mechanism of action is not very clear, and further studies will be needed to discriminate the roles of ARPC1B and WAS proteins in these two different mechanisms of DNA repair, we supposed that ARPC1B patients’ cells are able to trigger the DNA damage response, but they fail in its resolution, causing the maintenance of γH2AX. Moreover, ARPC1B localizes to centrosomes and interacts with Aurora-A kinase, influencing cell cycle progression, particularly in the G2/M-phase, and DNA end-resection in the HDR process, preventing mitotic entry (8, 25–27). Indeed, the negative effect of ARPC1B on Aurora-A activity has been clearly described by Molli et al. (8) to be responsible for delayed mitotic entry in ARPC1B-depleted cells (8, 27). However, in PtII-1 patients, γH2AX accumulation was particularly higher compared to the other ARPC1B patients, so we cannot exclude that other factors influence the RS trait.

Investigating the fate of damaged cells, we found a similar kinetic profile of cell survival during the 24 h of recovery culture in all tested samples, although with a cell line-specific rate before and after BLM treatment.

Of note, although a higher incidence of cancer has not been reported in ARPC1B patients so far, probably due to the early age of patients, our results encourage the need to monitor the disease evolution in these patients as in other defective HDR proteins that, albeit mildly affected, can lead to an increased risk of developing cancers (28–31).

## Concluding remarks

In conclusion, here we described, for the first time, an increased RS as a disease trait in ARPC1B deficiency. This trait can occur at different levels of severity depending on host factors that are still undefined. Further studies are needed to address the clinical relevance to designing specific clinical management and optimizing therapeutic interventions, including the choice of the HSCT conditioning regimen.

## Data availability statement

The datasets presented in this study can be found in online repositories. The names of the repository/repositories and accession number(s) can be found in the article/**Supplementary Material**.

## Ethics statement

The studies involving human participants were reviewed and approved by number:1702_OPBG_2018 and TIGET06, TIGET09. Written informed consent to participate in this study was provided by the participants’ legal guardian/next of kin.

## Author contributions

MC, GU, DA, NC, ANA, GD and CC interpreted the results and wrote the manuscript. MC, GU, FB, SP, CRC, MZ, SD, AB, FP, AC, VD’O, J-PV, MT, ANA and GD performed molecular and functional experiments and developed NGS analysis. CRC, AB, FP, AC, MT and GD created gene clusters to filter variants and integrated clinical and bioinformatics analysis of data retrieved by different genetic platforms. DA, NC, SV, IP, CG, EA, BR, LP, PZ, AGF, AD, GP, MG, MEH, AF, PP, PR, AA, and CC provided or referred clinical samples and patient’s clinical data. MC, GU, SV, MT, ANA, GD and CC participate to the study design and data interpretation. MC, GU, DA, NC, SV, MT, J-PV, AA, ANA, GD and CC made substantial contributions to revising the manuscript. ANA, GD, CC supervised the research and manuscript revision. All authors contributed to the article and approved the submitted version of manuscript.

## Funding

This work was funded by the Italian Ministry of Health (CCR-2017-23669081 and RCR-2020-23670068_001 to MT; NET-2011-02350069 to CC and AA; and RRC-2018-2365812 to CC), the Italian TELETHON Foundation (grant number GGP15109 to AF), Fondazione Bambino Gesù (Vite Coraggiose to MT), and the Italian Ministry of Research (FOE 2019 to MT) 2021_05_INFETT_CANCRINI to CC.

## Acknowledgments

The authors acknowledge the Rare Immunodeficiency, AutoInflammatory and AutoImmune Disease (RITA) network. The authors are grateful to patients and their families for participating in this study. We also thank Dr. Paola Ariganello for clinical assistance and our senior nurse Nadia Iavarone. We thank Jennifer Faudella and Patrizia Antimi for administrative assistance.

## Conflict of interest

The authors declare that the research was conducted in the absence of any commercial or financial relationships that could be construed as a potential conflict of interest.

The reviewer VB declared a shared affiliation with the author J-PV to the handling editor at the time of review.

## Publisher’s note

All claims expressed in this article are solely those of the authors and do not necessarily represent those of their affiliated organizations, or those of the publisher, the editors and the reviewers. Any product that may be evaluated in this article, or claim that may be made by its manufacturer, is not guaranteed or endorsed by the publisher.

## Supplementary material

Supplementary Figure 1
**(A)** Family pedigree showing proband (II1), carrier parents (I1, I2) and sister (II2). **(B)** Dermatological manifestations: blepharitis, papular and macular lesions of the face (a); dryness and lichenification on the upper arm and papular lesions on the trunk (b); flat warts on the dorsum of the right hand (c); erythematous plaques and erosions on the left axilla (d).

Supplementary Figure 2 Increased patient’s Treg cells. Frequency of CD4+CD25hiCD127lowFoxp3+ Treg cells, on the left, and Treg memory phenotype (CD45RA-) with Helios expression, on the right, from PtII-1 compared to an age-matched HD.

Supplementary Figure 3 Sanger sequencing characterization of PtII-1 and her family members. Sanger sequencing confirmed the homozygous deletion c.212_226del in ARPC1B gene (on the left panel). The red box in the ARPC1B wt sequence indicates the fifteen deleted nucleotides absent in Pt-II1 in which two inverted brackets show the break point; the overlapping ARPC1B sequence (black-wt and red-deleted) in I1, I2 and II2 indicates the carrier status. The right panel shows the homozygous mutation c.1606G>A in the SLC6A19 gene. The red point in the SLC6A19 sequence indicates the substituted nucleotide and double picks indicate the carrier status.

Supplementary Figure 4
**(A)** FACS analysis of ARPC1B protein expression on T-, B-, NK- cells and monocytes; the gate indicates the ARPC1B positive percentage relative to PtII-1. FMO means ‘fluorescence minus one’. **(B)** Western Blot of ARPC1B protein performed on EBVB and PHA-derived T cell lines. **(C)** showed the percentage of migrated PHA T-cells after 3 hours of stimulation with increasing concentrations of SDF1-α evaluated in PtII-1 and Pts ARPC1B (Pt2 and Pt3). Column bar graph shows mean ± standard error mean (SEM).

Supplementary Figure 5 Radio-sensitive studies: the upper panel shows primary fibroblasts stained with γH2AX after irradiation with 1 Gy and the lower panel shows the chromosomal sensitivity of EBVB cells treated in the G2-phase of the cell-cycle with 30 cGy X-rays. Statistical significance was evaluated with Student’s t-test: *p < 0.05; **p < 0.01.

Supplementary Figure 6 T Va7.2+/CD161- subset cells and TCRa signature in PtII-1’ and parents’ cells. **(A)**-up FACS analysis of the TCR-Va7.2 expression on CD3+ cells from patient (on the right), mother (in the middle) and father (on the left); **(A)**-down shows the measure of the naive T cell subset (CD3+CD4+CD197+CD45RA+) in patient and her parents. **(B)** Unsupervised hierarchical clustering analysis (upper) of PROMIDISα data shows PtII-1 clustered closer to V(D)J and AT patient groups, while her DAD (I-1) and MUM (I-2) nearer to healthy controls (WT) group. The derived dendrogram (lower) of the TCRα repertoire analysis depicts PtII-1, pointed by the red arrow, within the V(D)J group while her parents, pointed by the grey arrows, into the WT group (Ctl).

Supplementary Figure 7 Radiosensitivity and cytoskeleton characterization of Pt3 fibroblasts. Confocal representative images of F-actin (red) and γH2AX (green) expression with nuclei counterstained in blue on primary fibroblasts obtained from Pt3-ARPC1B and HD, w/o or with BLM treatment (9µM/1h) and a repair incubation time (4 hours).

## Abbreviations

ARPC1B, actin-related protein 2/3 complex subunit 1B; IEI, inborn errors of immunity; HSCT, hematopoietic stem cell transplantation; Arp2/3, actin-related protein 2/3; DDR, DNA damage response; CID, combined immunodeficiency; WASp, Wiskott–Aldrich syndrome protein; HDR, homology-directed repair; GDR, Golgi-dispersal response; ATM, ataxia telangiectasia; NBS, Nijmegen breakage syndrome; DSBs, double-strand breaks; NGS, next-generation sequencing; WES, whole-exome sequencing; WGS, whole-genome sequencing; EBVB, Epstein–Barr virus-infected B cells; γH2AX, phosphorylated histone H2AX; NHEJ, nonhomologous end joining; IR, ionizing radiation; BLM, bleomycin.

## References

[1] Kahr WH Pluthero FG Elkadri A Warner N Drobac M Chen CH. Loss of the Arp2/3 complex component ARPC1B causes platelet abnormalities and predisposes to inflammatory disease. Nat Commun (2017) 8:14816. doi: 10.1038/ncomms14816 28368018 PMC5382316

[2] Kuijpers TW Tool ATJ van der Bijl I de Boer M van Houdt M de Cuyper IM. Combined immunodeficiency with severe inflammation and allergy caused by ARPC1B deficiency. J Allergy Clin Immunol (2017) 140(1):273–7. doi: 10.1016/j.jaci.2016.09.061 27965109

[3] Somech R Lev A Lee YN Simon AJ Barel O Schiby G. Disruption of thrombocyte and T lymphocyte development by a mutation in ARPC1B. J Immunol (2017) 199(12):4036–45. doi: 10.4049/jimmunol.1700460 PMC5726601 29127144

[4] Brigida I Zoccolillo M Cicalese MP Pfajfer L Barzaghi F Scala S. T-Cell defects in patients with ARPC1B germline mutations account for combined immunodeficiency. Blood (2018) 132(22):2362–74. doi: 10.1182/blood-2018-07-863431 PMC6265646 30254128

[5] Volpi S Cicalese MP Tuijnenburg P Tool ATJ Cuadrado E Abu-Halaweh M. A combined immunodeficiency with severe infections, inflammation, and allergy caused by ARPC1B deficiency. J Allergy Clin Immunol (2019) 143(6):2296–9. doi: 10.1016/j.jaci.2019.02.003 PMC6677392 30771411

[6] Papadatou I Marinakis N Botsa E Tzanoudaki M Kanariou M Orfanou I. Case report: A novel synonymous ARPC1B gene mutation causes a syndrome of combined immunodeficiency, asthma, and allergy with significant intrafamilial clinical heterogeneity. Front Immunol (2021) 12:634313. doi: 10.3389/fimmu.2021.634313 33679784 PMC7933039

[7] Schrank BR Aparicio T Li Y Chang W Chait BT Gundersen GG. Nuclear ARP2/3 drives DNA break clustering for homology-directed repair. Nature (2018) 559(7712):61–6. doi: 10.1038/s41586-018-0237-5 PMC6145447 29925947

[8] Molli PR Li DQ Bagheri-Yarmand R Pakala SB Katayama H Subrata SA. Arpc1b, a centrosomal protein, is both an activator and substrate of aurora a. J Cell Biol (2010) 190(1):101–14. doi: 10.1083/jcb.200908050 PMC2911675 20603326

[9] German Y Vulliard L Kamnev A Pfajfer L Huemer J Mautner AK. Morphological profiling of human T and NK lymphocytes by high-content cell imaging. Cell Rep (2021) 36(1):109318. doi: 10.1016/j.celrep.2021.109318 34233185

[10] Wen KK Han SS Vyas YM. Wiskott-Aldrich syndrome protein senses irradiation-induced DNA damage to coordinate the cell-protective golgi dispersal response in human T and b lymphocytes. J Allergy Clin Immunol (2020) 145(1):324–34. doi: 10.1016/j.jaci.2019.09.026 PMC6949418 31604087

[11] Bröer S. The role of the neutral amino acid transporter B0AT1 (SLC6A19) in hartnup disorder and protein nutrition. IUBMB Life (2009) 61(6):591–9. doi: 10.1002/iub.210 PMC7165679 19472175

[12] Berland A Rosain J Kaltenbach S Allain V Mahlaoui N Melki I. PROMIDISα: A T-cell receptor α signature associated with immunodeficiencies caused by V(D)J recombination defects. J Allergy Clin Immunol (2019) 143(1):325–34.e2. doi: 10.1016/j.jaci.2018.05.028 29906526

[13] Mladenov E Fan X Dueva R Soni A Iliakis G. Radiation-dose-dependent functional synergisms between ATM, ATR and DNA-PKcs in checkpoint control and resection in G2-phase. Sci Rep (2019) 9:8255. doi: 10.1038/s41598-019-44771-6 31164689 PMC6547644

[14] Leung G Zhou Y Ostrowski P Mylvaganam S Boroumand P Mulder DJ. ARPC1B binds WASP to control actin polymerization and curtail tonic signaling in b cells. JCI Insight (2021) 6(23):e149376. doi: 10.1172/jci.insight.149376 34673575 PMC8675194

[15] Nahasa SA Gatti RA. DNA Double strand break repair defects, primary immunodeficiency disorders, and ‘radiosensitivity’. Curr Opin Allergy Clin Immunol (2009) 9:510–6. doi: 10.1097/ACI.0b013e328332be17 19858715

[16] Grossberg AL. Update on pediatric photosensitivity disorders. Curr Opin Pediatr (2013) 25(4):474–9. doi: 10.1097/MOP.0b013e328362c2c3 23817304

[17] Moulding DA Record J Malinova D Thrasher AJ. Actin cytoskeletal defects in immunodeficiency. Immunol Rev (2013) 256(1):282–99. doi: 10.1111/imr.12114 PMC3884764 24117828

[18] Tangye SG Bucciol G Casas-Martin J Pillay B Ma CS Moens L. Human inborn errors of the actin cytoskeleton affecting immunity: way beyond WAS and WIP. Immunol Cell Biol (2019) 97(4):389–402. doi: 10.1111/imcb.12243 30779216

[19] Papa R Penco F Volpi S Gattorno M. Actin remodeling defects leading to autoinflammation and immune dysregulation. Front Immunol (2020) 11:604206. doi: 10.3389/fimmu.2020.604206 33488606 PMC7817698

[20] Sprenkeler EJJ Webbers SDS Kuijpersa TW. When actin is not actin’ like it should: A new category of distinct primary immunodeficiency disorders. J Innate Immun (2021) 13(1):3–25. doi: 10.1159/000509717 32846417 PMC7879252

[21] Rothkamm K Horn S. Gamma-H2AX as protein biomarker for radiation exposure. Ann Ist Super Sanita (2009) 45(3):265–71. 19861731

[22] Goodarzi AA Jeggo PA. Irradiation induced foci (IRIF) as a biomarker for radiosensitivity. Mutat Res (2012) 736(1-2):39–47. doi: 10.1016/j.mrfmmm.2011.05.017 21651917

[23] Natarajan AT Obe G Hayashi M. Chromosomal aberrations. Mutat Res (2008) 657(1):1–2. doi: 10.1016/j.mrgentox.2008.08.013 18793751

[24] Scarpato R Castagna S Aliotta R. Kinetics of nuclear phosphorylation (γ-H2AX) in human lymphocytes treated *in vitro* with UVB, bleomycin and mitomycin c. Mutagenesis (2013) 28(4):465–73. doi: 10.1093/mutage/get024 23696313

[25] Bednarski JJ Sleckman BP. Integrated signaling in developing lymphocytes: the role of DNA damage responses. Cell Cycle (2012) 11(22):4129–34. doi: 10.4161/cc.22021 PMC3524208 23032308

[26] Byrum AK. Mitotic regulators TPX2 and aurora a protect DNA forks during replication stress by counteracting 53BP1 function. J Cell Biol (2019) 218:422–32. doi: 10.1083/jcb.201803003 PMC6363440 30602538

[27] Ma HT. Poon RYC aurora kinases and DNA damage response. Mutat Res (2020) 821:11171. doi: 10.1016/j.mrfmmm.2020.111716 32738522

[28] El-Nachef L Al-Choboq J. Human radiosensitivity and radiosusceptibility: What are the differences? Int J Mol Sci (2021) 22(13):7158. doi: 10.3390/ijms22137158 34281212 PMC8267933

[29] Ghosal G Chen J. DNA Damage tolerance: a double-edged sword guarding the genome. Transl Cancer Res (2013) 2(3):107–29. doi: 10.3978/j.issn.2218-676X.2013.04.01 PMC3779140 24058901

[30] Campos A Clemente-Blanco A. Cell cycle and DNA repair regulation in the damage response: Protein phosphatases take over the reins. Int J Mol Sci (2020) 21(2):446. doi: 10.3390/ijms21020446 PMC7014277 31936707

[31] van Gijn SE Wierenga E. TPX2/Aurora kinase a signaling as a potential therapeutic target in genomically unstable cancer cells. Oncogene (2019) 38:852–67. doi: 10.1038/s41388-018-0470-2 PMC6367211 30177840

