# Supplementary figures and images for: Radiosensitivity in patients affected by ARPC1B deficiency: a new disease trait?
Source: Front Immunol. 2022 Jul 29;13:919237. doi: 10.3389/fimmu.2022.919237 (PMC9372879; doi:10.3389/fimmu.2022.919237)

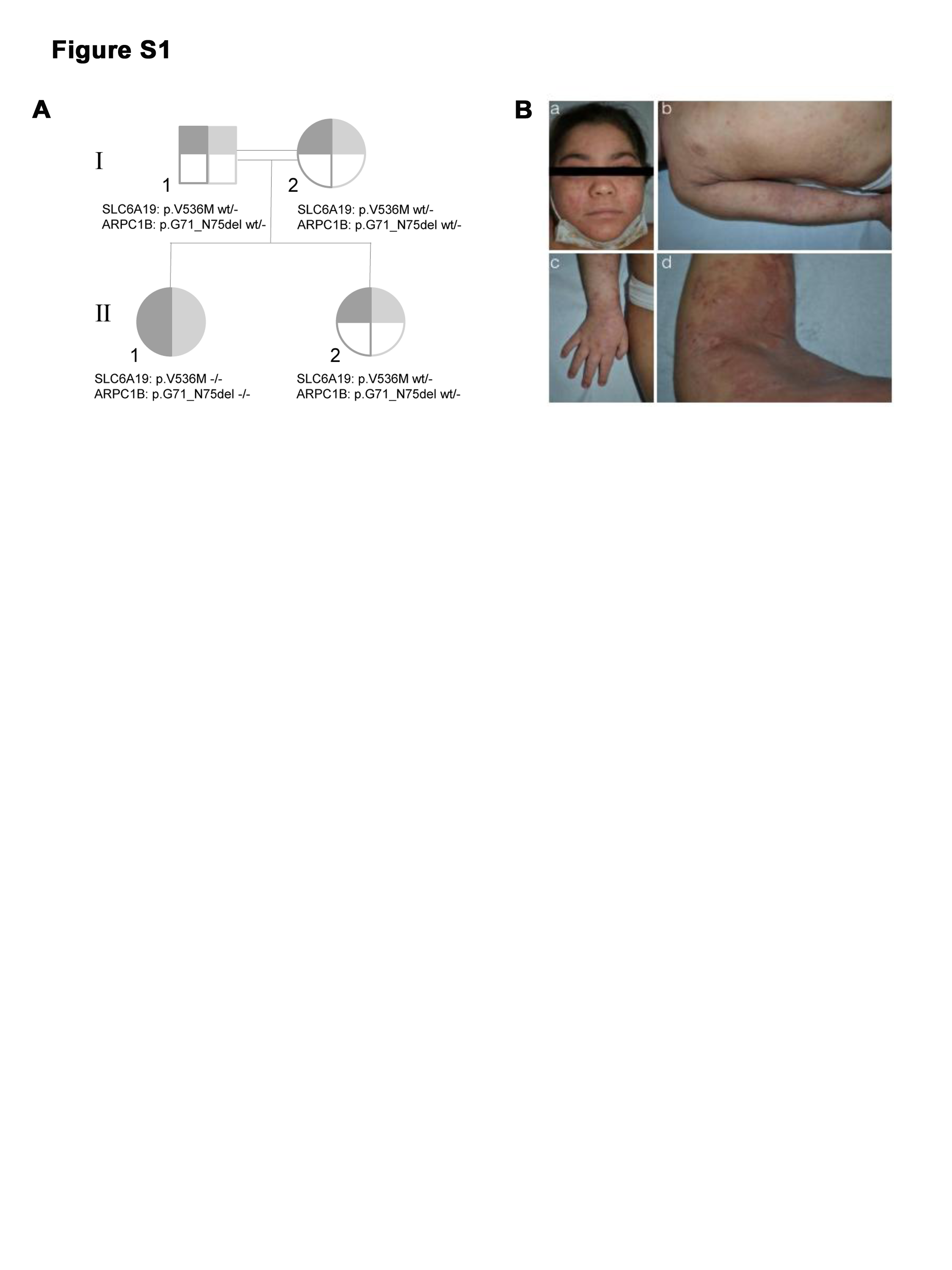

Supplement: Supplementary Figure 1 — (A) Family pedigree showing proband (II1), carrier parents (I1, I2) and sister (II2). (B) Dermatological manifestations: blepharitis, papular and macular lesions of the face (a); dryness and lichenification on the upper arm and papular lesions on the trunk (b); flat warts on the dorsum of the right hand (c); erythematous plaques and erosions on the left axilla (d). [file Image_1.tif]

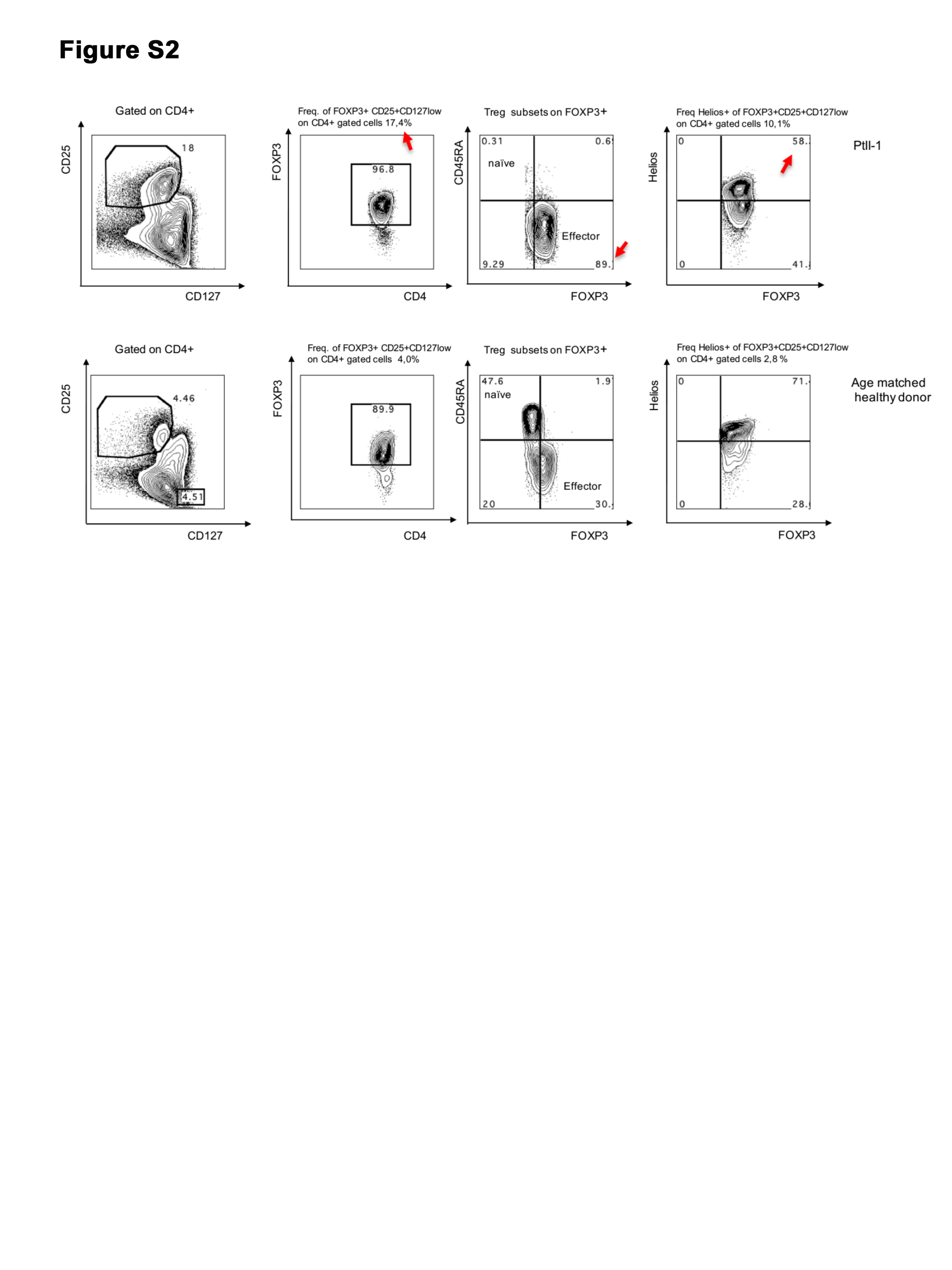

Supplement: Supplementary Figure 2 — Increased patient’s Treg cells. Frequency of CD4+CD25hiCD127lowFoxp3+ Treg cells, on the left, and Treg memory phenotype (CD45RA-) with Helios expression, on the right, from PtII-1 compared to an age-matched HD. [file Image_2.tif]

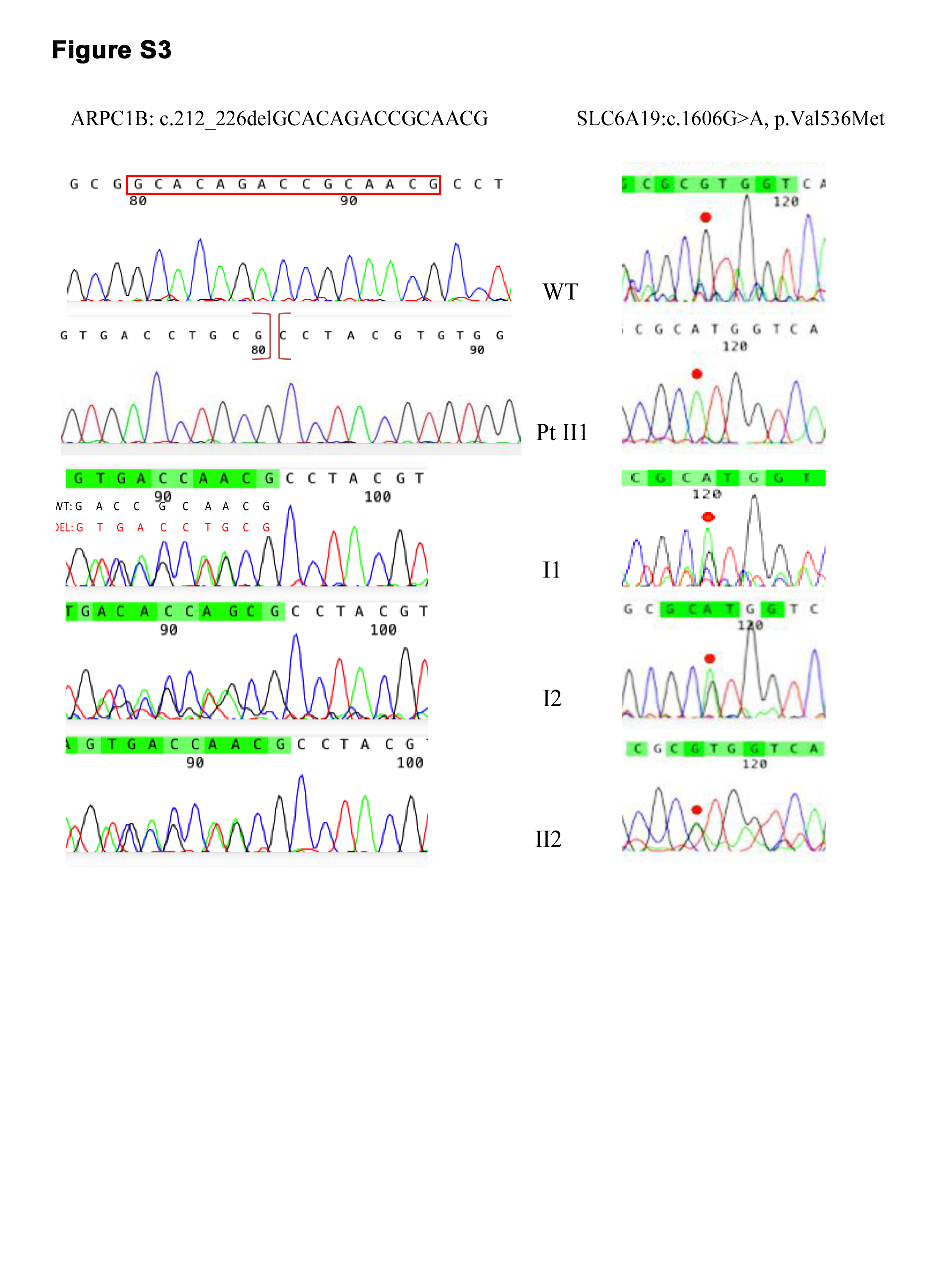

Supplement: Supplementary Figure 3 — Sanger sequencing characterization of PtII-1 and her family members. Sanger sequencing confirmed the homozygous deletion c.212_226del in ARPC1B gene (on the left panel). The red box in the ARPC1B wt sequence indicates the fifteen deleted nucleotides absent in Pt-II1 in which two inverted brackets show the break point; the overlapping ARPC1B sequence (black-wt and red-deleted) in I1, I2 and II2 indicates the carrier status. The right panel shows the homozygous mutation c.1606G>A in the SLC6A19 gene. The red point in the SLC6A19 sequence indicates the substituted nucleotide and double picks indicate the carrier status. [file Image_3.tif]

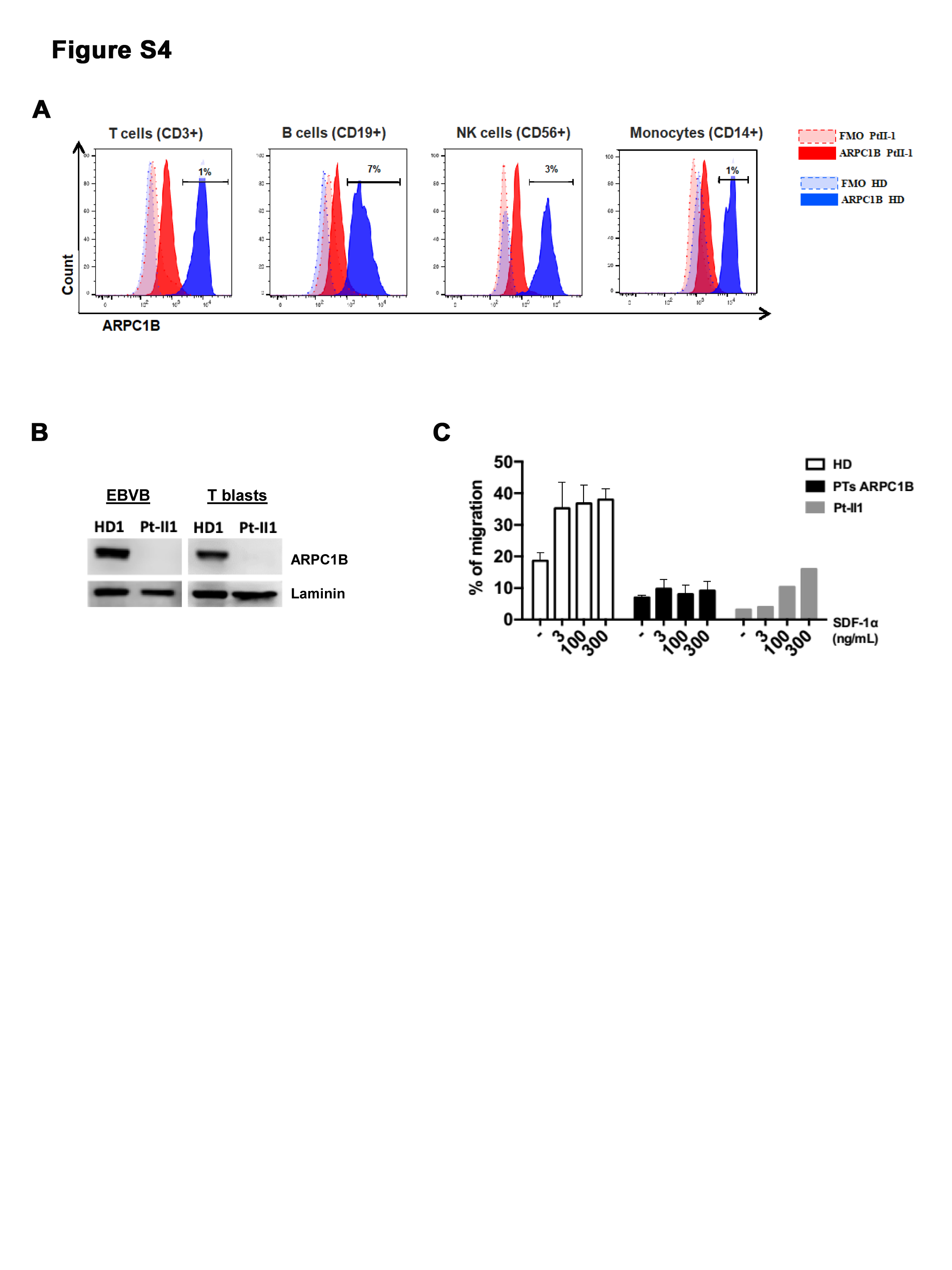

Supplement: Supplementary Figure 4 — (A) FACS analysis of ARPC1B protein expression on T-, B-, NK- cells and monocytes; the gate indicates the ARPC1B positive percentage relative to PtII-1. FMO means ‘fluorescence minus one’. (B) Western Blot of ARPC1B protein performed on EBVB and PHA-derived T cell lines. (C) showed the percentage of migrated PHA T-cells after 3 hours of stimulation with increasing concentrations of SDF1-α evaluated in PtII-1 and Pts ARPC1B (Pt2 and Pt3). Column bar graph shows mean ± standard error mean (SEM). [file Image_4.tif]

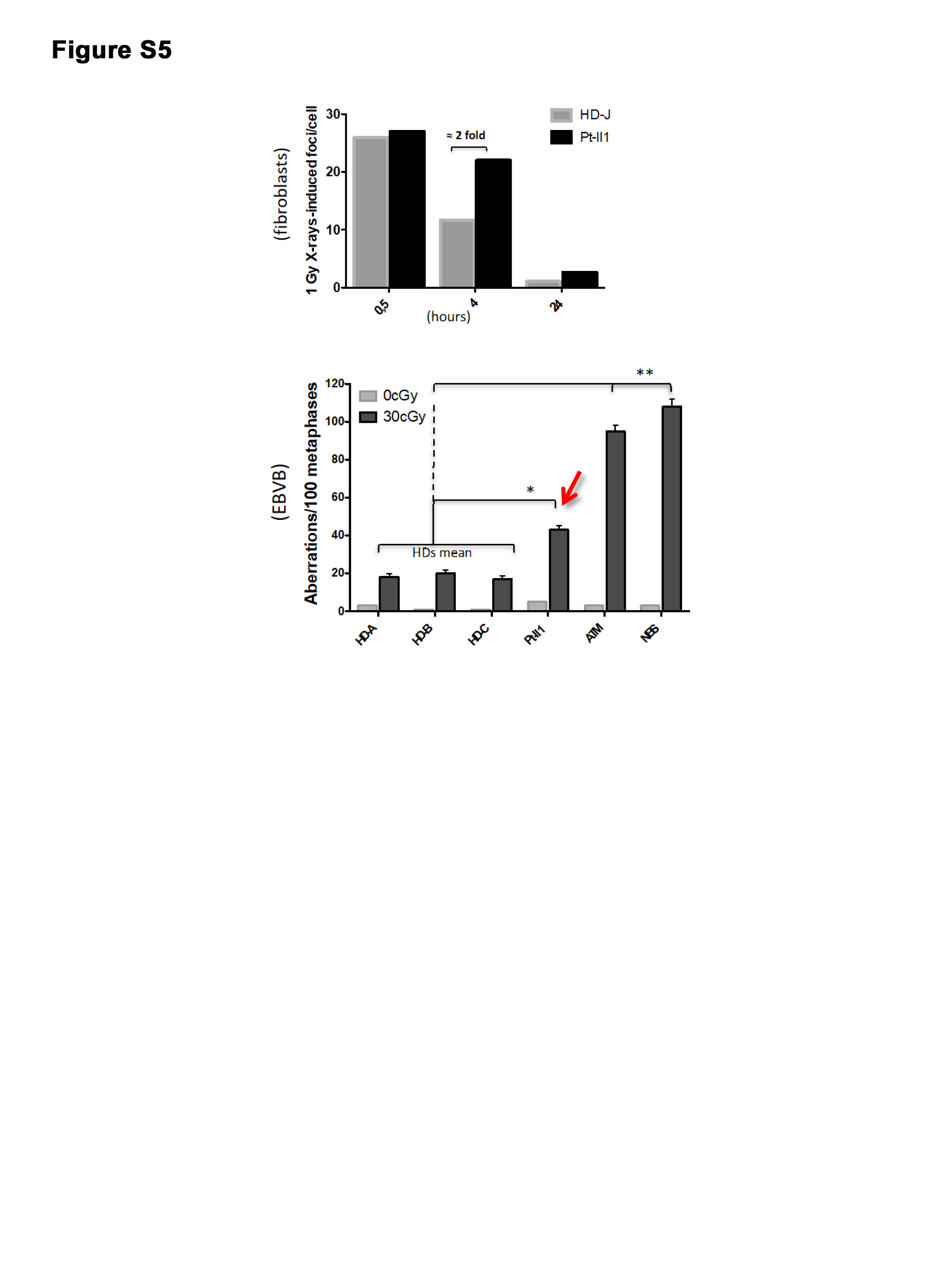

Supplement: Supplementary Figure 5 — Radio-sensitive studies: the upper panel shows primary fibroblasts stained with γH2AX after irradiation with 1 Gy and the lower panel shows the chromosomal sensitivity of EBVB cells treated in the G2-phase of the cell-cycle with 30 cGy X-rays. Statistical significance was evaluated with Student’s t-test: *p < 0.05; **p < 0.01. [file Image_5.tif]

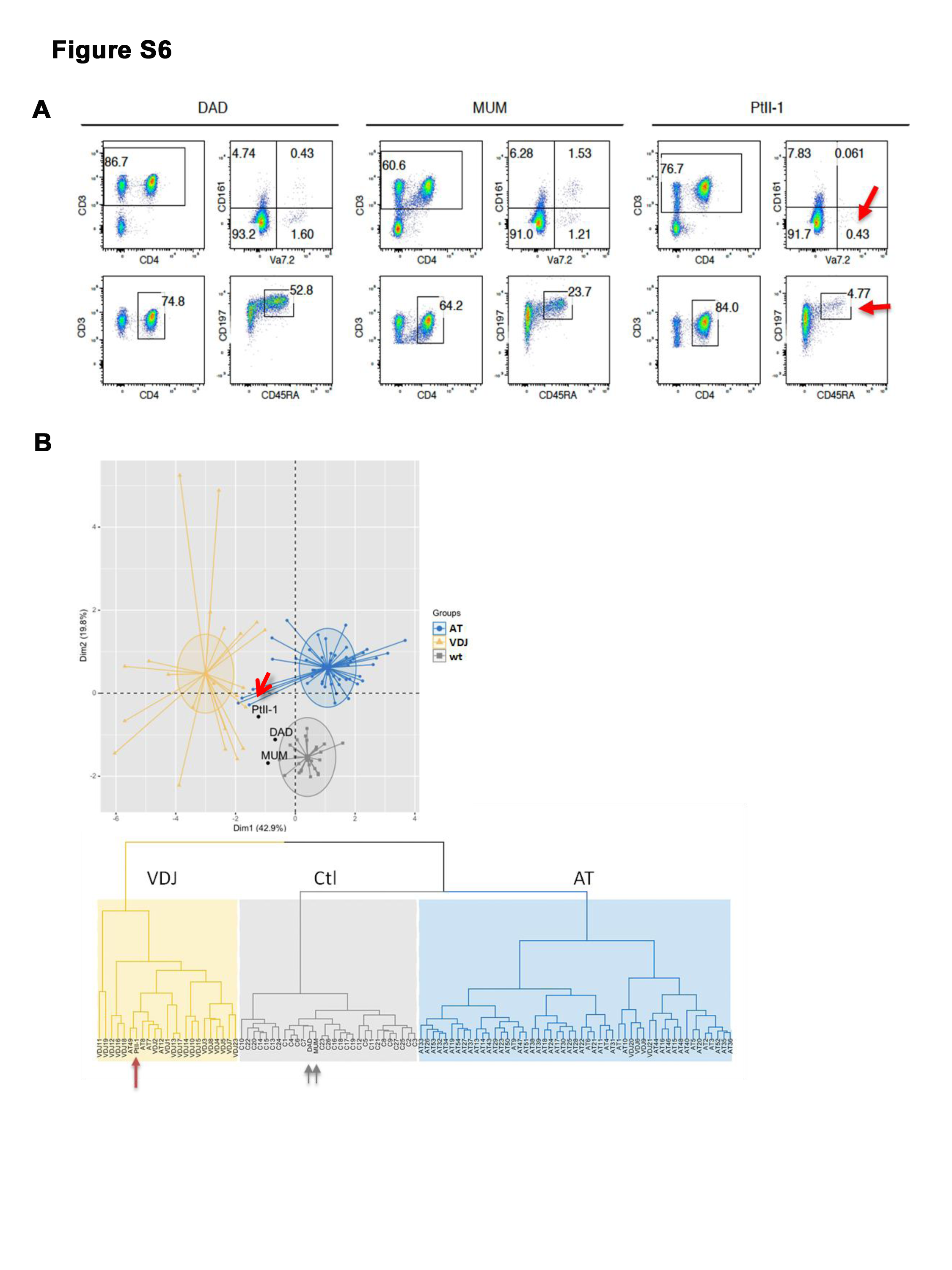

Supplement: Supplementary Figure 6 — T Va7.2+/CD161- subset cells and TCRa signature in PtII-1’ and parents’ cells. (A)-up FACS analysis of the TCR-Va7.2 expression on CD3+ cells from patient (on the right), mother (in the middle) and father (on the left); (A)-down shows the measure of the naive T cell subset (CD3+CD4+CD197+CD45RA+) in patient and her parents. (B) Unsupervised hierarchical clustering analysis (upper) of PROMIDISα data shows PtII-1 clustered closer to V(D)J and AT patient groups, while her DAD (I-1) and MUM (I-2) nearer to healthy controls (WT) group. The derived dendrogram (lower) of the TCRα repertoire analysis depicts PtII-1, pointed by the red arrow, within the V(D)J group while her parents, pointed by the grey arrows, into the WT group (Ctl). [file Image_6.tif]

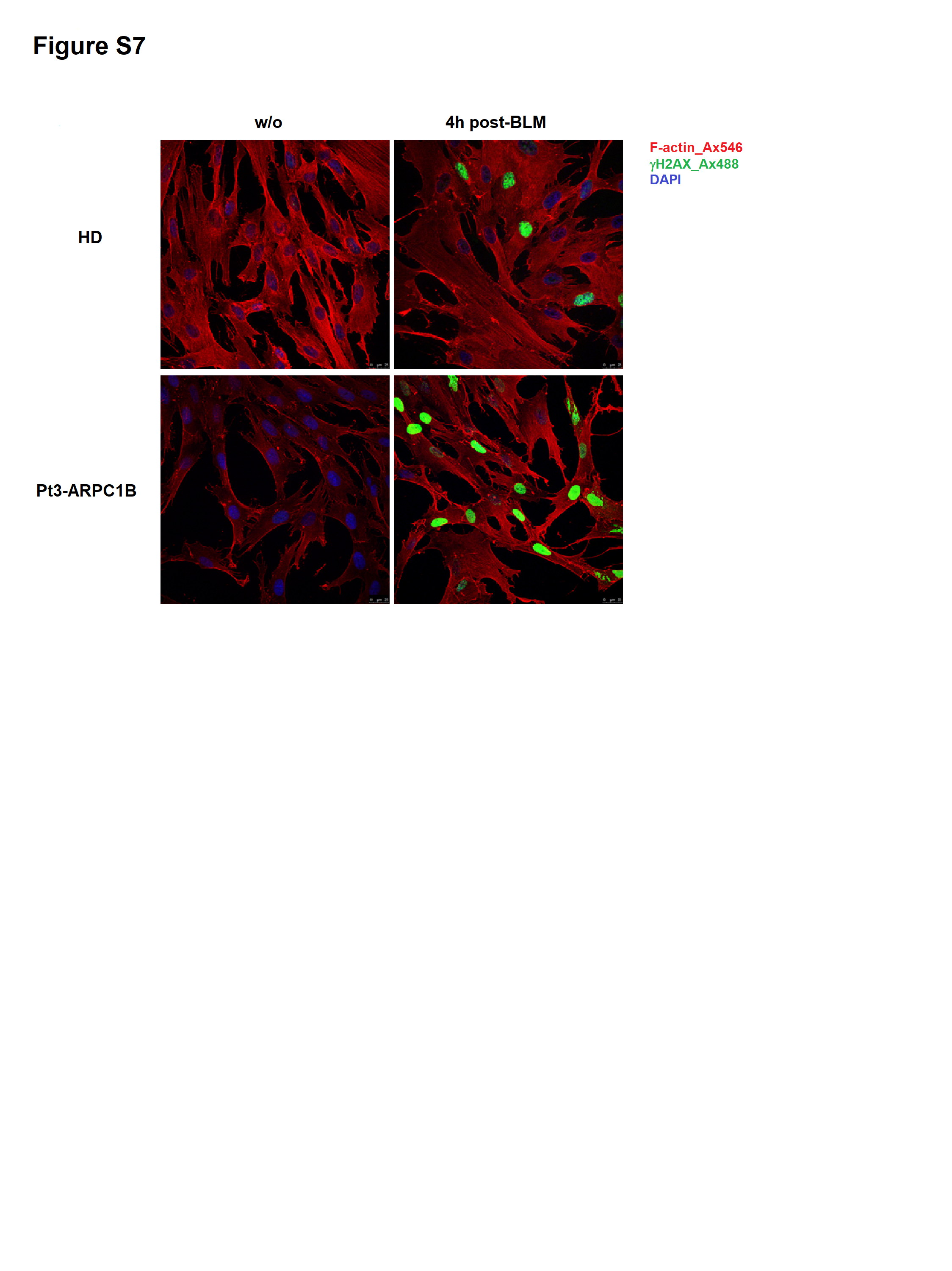

Supplement: Supplementary Figure 7 — Radiosensitivity and cytoskeleton characterization of Pt3 fibroblasts. Confocal representative images of F-actin (red) and γH2AX (green) expression with nuclei counterstained in blue on primary fibroblasts obtained from Pt3-ARPC1B and HD, w/o or with BLM treatment (9µM/1h) and a repair incubation time (4 hours). [file Image_7.tif]
