# Supplementary material for: Radiosensitivity in patients affected by ARPC1B deficiency: a new disease trait?
Source: Front Immunol. 2022 Jul 29;13:919237. doi: 10.3389/fimmu.2022.919237 (PMC9372879; doi:10.3389/fimmu.2022.919237)
Supplement: Supplementary file 8 [file DataSheet_1.docx]

**SUPPLEMENTARY MATERIALS**

**Characterization of case-index PtII-1**

*Therapy*

Since the age of 2 years and 10 months she received oral corticosteroid therapy with a good clinical response on recurrent infections and dermatitis. After 9 months, the corticosteroid treatment was tapered and later interrupted due to EBV and HHV-6 infections and blepharitis (S. Aureus). After steroid interruption she suffered from severe and persistent dermatitis together with hemorrhagic diarrhea requiring hospital admission. A gastrointestinal endoscopy and colonoscopy revealed the presence of IBD-like aphthous lesions. This evidence led us to begin a combination of treatments with different anti-inflammatory and immunosuppressive drugs. Chronic inflammation was initially treated with Anakinra (anti-IL1R) that temporarily improved the eczema but it was stopped because of the appearance of skin abscess. She then started Mesalazine, Azathioprine and endorectal Beclomethasone improving gastrointestinal and dermatitis symptoms that relapsed later. To solve recurrent cutaneous abscesses and relapsing dermatitis, she started mycophenolate-mofetil with a partial improvement of the skin lesions, but few months later reduced because of the development of warts positive for HPV-14 on both ears and perianal area, and worsening of thrombocytopenia. Topic imiquimod partially ameliorated the warts, and treatment with rituximab, everolimus and subcutaneous Ig controlled partially both dermatitis and infections.

**Methods concerning molecular, cellular and functional investigations**

*Molecular investigation*

External Companies performed Whole Exome Sequencing (WES) and Whole Genome Sequencing (WGS) (<https://www.genewiz.com/en-GB/Public/Services/Next-Generation-Sequencing/>). gDNA was isolated from total PBMCs for Sanger sequencing. The mutated exons of the ARPC1B (NCBI NM_005720.4) and SLC6A19 (NCBI NM_001003841.3) genes were amplified by PCR (GoTaq Polimerase-Promega), sequenced using the BigDye Terminator version 3.1 Cycle Sequencing Kit (Applied Biosystems, Foster City, CA) and analyzed on an ABI PRISM 3130 and 310 automated sequencers (Applied Biosystems, Foster City, CA).

Trio-based WES analysis: exome capture was carried out using SureSelect Clinical Research Exome v2 (Agilent) and sequencing was performed on a NextSeq550 platform. WES raw data were processed and analyzed using a previously described in-house implemented pipeline (1-2), which is based on the GATK Best Practices (3). Reads were aligned to RCh37/hg19 by using the BWA-MEM tool (4). Variant calling was attained with HaplotypeCaller (GATK v3.7) (3). We used the SnpEff v.4.3 and dbNSFP v.3.5 tools (5-6) for functional annotation of variants, including CADD v.1.3, M-CAP v.1.0 and Intervar v.0.1.6 for functional impact prediction (7-9). Thereby, the analysis was narrowed to variants, which affect coding sequences or splice site regions. High-quality variants were filtered against public (dbSNP150 and gnomAD V.2.0.1, threshold 0.1%) and in house (threshold 1%, ~2000 population-matched exomes) databases. WES statistics and data output is reported in Table S2.

Trio-based WGS analysis: an in-house implemented pipeline based on GATK Best Practices was used (3). Briefly, raw reads were aligned to the GRCh38 human reference genome, excluding centromeric regions, using BWA-MEM (v0.7.12) (4). Short variants (SNPs and Indels) were then jointly called by means of HaplotypeCaller and GenotypeGVCFs algorithms (3) and phased. High quality variants were filtered with the VQSR tool, setting the sensitivity thresholds to maximize sensitivity of variant detection in comparison with WES data previously performed on the same samples. A median coverage of 19x was obtained, with 90% of genome covered >10x. WGS and variant calling metrics are summarized in table S2. Variants within coding regions were annotated as above (Table S3). Variants in non-coding regions were annotated and prioritized with Genomiser (10). Structural variants (> 50-bp-long) were called and genotyped using DELLY v8.2 with default parameters annotated and prioritized with AnnotSV (11-12).

*Cell culture*

Human PBMC were isolated from patients and healthy controls (HDs) included in the study by density-gradient centrifugation with Ficoll-Paque PLUS (GE Healthcare) according to the manufacturer’s protocol. PBMC were cultured in complete medium (RPMI-1640, 10% FBS, glutamine, penicillin and streptomycin) and to generate T cell lines, cells were activated with PHA (1 μg/ml, SIGMA) and IL2 (100 IU/ml, SIGMA) or to generate EBVB cell lines plated 2h (37°C/5% CO2) in B95-8 EBV supernatant (0.22 μm filtered) and then plated in RPMI 20% FBS. After 24h 20% FBS complete medium with 5mg/ml cyclosporine was added. Cells were cultured for 2 weeks to generate EBVB clumps.

*Western Blot*

ARPC1B investigation: Total protein lysates were obtained from fibroblasts, EBVB and T cell lines from patients and HD using compete JS 1X lysis buffer (50 mM Tris/HCl ph 8, 150 mM NaCl, 1.5 mM MgCl2, 5 mM EGTA, 1% Triton-X, 10% glycerol, 1 mM PMSF, aprotinin 1 mg/ml, leupeptin 1 mg/ml, pepstatin 1 mg/ml, 1 mM DTT) for 20 minutes on ice and then clarified by centrifugation at 1600 rpm for 10 min at 4°C. Lysates were size-fractionated by SDS-PAGE gel (12%) and then transferred to nitrocellulose membrane (Protran by Schleicher & Schuell-Bioscience, Dassel, Germany). Membrane was blocked in 5% BSA for 1 h at room temperature and then incubated with ARPC1B antibody (1h 1:500, ab99314-rabbit-Abcam)/goat anti-rabbit IgG (1h/RT, Cell Signaling) and then β-actin (1h 1:5000, SIGMA)/ goat anti-mouse IgG (1h/RT, Cell Signaling).

*Migration assay*

Total PBMCs were seeded on a transwell chamber in presence of scalar dilutions of SDF1-α (Peprotech) and migration capacity was assessed after 3 hours. PHA T-cell blasts used for the detection of ARPC1B before and after migration were plated at the concentration of 1x106 T cells/condition. T cell blasts were stained for ARPC1B expression before migration as a quantification of input of ARPC1B+ cells. After migration, migrated cells were harvested, counted and stained for ARPC1B and CD8 as reported. 30.000 events were acquired in the lymphogate and analyzed by FlowJo software. Absolute numbers of ARPC1B+CD8+ and ARPC1B-CD8+ were calculated with the following formula: [(%ARPC1B+CD8+ or %ARPC1B-CD8+/100)*(nr migrated CD8+ cells)].

*FACS studies*

All data results were acquired on FACSCanto II (Becton Dickinson) and analyzed with FlowJo software (Tree Star Inc, version 9.3.2).

Immunophenotype: All flow cytometric analyses were performed on EDTA blood samples within 24 h of venipuncture. After red blood cell lysis with ammonium chloride, lymphocytes were washed, resuspended in PBS, and stained with the following mouse antihuman antibodies to identify T and B cell subsets: CD45RA APCH7 (clone T6D11; Miltenyi Biotec), CD3 PerCP (clone BW264/56; Miltenyi Biotec), CCR7 PE (clone 3D12; eBioscience), CD4 APC (clone OKT4; Becton Dickinson), CD8 PE-Cy7 (clone RPA-T8; Becton Dickinson), CD19 PE-CY7 (clone SJ25C1; Becton Dickinson), CD16 PE (clone 3G8), CD56 PE, CD27 FITC (cloneM-T271, Becton Dickinson), TCR α-beta APC (clone T10B9; Becton Dickinson), TCR gamma-delta FITC (11F3; Miltenyi Biotec), CD21 PE (clone B-ly4; Becton Dickinson), CD24 PE (clone ML5; Becton Dickinson), IgD FITC (clone IA6-2; Becton Dickinson), Goat F(ab) 2 anti-Human IgM (μ)-Alexa Fluor 647 (Jackson ImmunoResearch), and CD38 FITC (clone HIT2; Becton Dickinson). Cells were incubated with the appropriate antibody cocktail for 30 min at 4°C and then washed with PBS and resuspended in PBS for flow cytometric acquisition. At least 50,000 events were acquired within the lymphogate.

Regulatory T Cells: For surface staining, cells were pre-treated with Fc-blocking reagent (eBioscience) and then surface staining antibodies were added and incubated for 20 min 4°C. The following Abs were used: CD4 (APC, OKT4) (Becton Dickinson), FoxP3 (AlexaFluor488, PHC101), Helios (PE-CY7, 22F6) and CD127 (PE-Cy7, ebioRDR5) from ebioscience, CD45RA APC-VIO770 (HI100) from Miltenyi. Intracellular stainings with FoxP3 and Helios were performed according to manufacturer’s instructions with FoxP3 Fixation/Permeabilization Buffer (eBioscience). At least 200,000 CD4+ events within lymphocyte gate were acquired for each sample.

ARPC1B expression: Total PBMCs (300.000 per tube) were stained for CD3, CD4, CD8, CD19, CD14, CD56 lymphocytes surface markers (BD Bioscience) and intracellular staining for ARPC1B was performed after fixation and permeabilization (Cytofix/Cytoperm kit, BD Biosciences) according to manufacturer’s protocol. Polyclonal rabbit anti human-ARPC1B antibody (Abcam, ab99314) was used at 1:50. After 30 min of incubation at room temperature cells were washed 3 times, stained with goat-anti-Rabbit Alexa 488 in permeabilization buffer and incubated for 30 min at 4°C. An additional incubation in PBS-FACS for 30 min at 4°C was done to avoid any aspecific staining. Surface staining was performed for 20 min at 4°C.

T-cell receptor Vα7.2 and naïve T cells determination: 100 µL of whole blood was incubated for 30 minutes at 4°C with 5 µL of both indicated fluorochrome-labeled mAbs: allophycocyanin anti-CD3 (HIT3a; Sony, Weybridge, United Kingdom) and phycoerythrin anti-TCR Vα7.2 (3C10; Sony). After red cell lysis (FACS Lysing Solution; BD, Franklin Lakes, NJ) and washing of the remaining cells twice with cell wash buffer (BD), cells were fixed in a cell fixation solution (BD). CD3^+^ cells were analyzed by means of flow cytometry within 24 hours (FACSCanto II; BD). Expression of TCR Vα7.2 (the most distal/upstream T-cell receptor α variable segment, TRAV1) on CD3^+^ lymphocytes was assessed by using FlowJo software (Tree Star) [27]. Additionally, 100 µL of whole blood was incubated with four fluorochrome-labeled mAbs from the *Human Naive/Memory T cell ID Panel* (Sony, #2411005) according to the manufacturer’s protocol. The naive T cells were identified through CD45RA^+^/CD197^+^ cells amongst the CD3^+^/CD4^+^ using FlowJo software (Tree Star).

*PROMIDISα analysis:* cDNA from PBMC was amplified with a series of 8 primers specific for Hu-TRAV segments representing proximal (TRAV35 and TRAV41), middle (TRAV20, TRAV21, and TRAV23), and distal (TRAV1, TRAV5, and TRAV10) TRAV segments together with TRAC3-REPF, a Cα-specific primer. Primers include an anchor sequence (RepF and RepR) for subsequent NGS sequencing on the PGM. TCRα repertoire was analyzed by using PROMIDISα.

**Radiosensitivity Assays**

Irradiation: Epstein-Barr Virus B (EBVB) cell lines were exposed at room temperature to 30 cGy X-rays delivered by a Gilardoni MGL 300/6D apparatus operating at 250 kV, 6 mA). Cells were incubated with 5x10-6 M colchicine for 3 hours after irradiation, then harvested and chromosome preparations obtained by conventional methods. Hundred Giemsa-stained metaphases were scored for each experimental point in repeated independent experiments. For the scoring of γH2AX foci, fibroblasts grown on glass coverslips were exposed to 1 Gy and fixed at 30 min, 2, 4 and 24 hours from treatment for 10 min in 2% paraformaldehyde, permeabilized on ice for 5 min with 0.2% Triton X-100 and blocked in PBS/BSA 1% for 30 min. Slides were incubated with 1 ug/ml of γH2AX mouse monoclonal antibody and detected with an anti-mouse FITC conjugated antibody. After counterstaining with DAPI, images were captured using a Zeiss Axiophot epifluorescent microscopy equipped with a CCD camera. Quantitative analysis was performed counting by eye foci in at least 50 nuclei (13).

Bleomycin assay: EBVB cells were treated with chemical radiomimetic-induced DNA damage bleomycin (BLM) [9 µM, 1 h/37 °C] and then plated in complete RPMI (10% FBS) for 2 h at 37 °C to perform γH2AX-FACS or 4 h to perform γH2AX-IF studies; additional time points of incubation (4, 6, 8, 10, 24 hours) for DNA damage repair and cell viability were investigated.

*a) FACS analysis:* EBVB cells treated or not were collected and washed twice in PBS (SIGMA). Cell pellets were resuspended in ice-cold 70% EtOH and incubate over night at -20 °C. The next day cell pellets were washed twice in BSA-T-PBS buffer (1% BSA, 0,2% Triton X-100 in PBS), centrifuged and then stained in AlexaFluor488 mouse anti-γH2AX antibody (BD, 1:100, 20 min, 4 °C). After incubation, pellets were washed and resuspended in Propidium iodide [10 μg in PBS] and acquired by FACS Canto II.

*b) Immunofluorescence/Confocal Laser Microscopy:* 12-15 x 10^4^ EBVB cells treated or not were collected and washed twice with PBS. Cells in PBS were seeded and dried on positively-charged slides, then fixed in 4% paraformaldehyde (SIGMA) for 10 min. Cells were then washed with PBS and permeabilized for 10 min with PBS/0.1% Triton X-100. Cross-linking reactions were blocked by adding PBS/0.5% BSA (30 min). γH2AX expression on EBVB cells was detected by incubation of cells with AlexaFluor488 mouse anti-γH2AX antibody (BD Pharmangen: 560445) in a humidified chamber for 1 h at 37 °C. Nuclei were counterstained with Hoechst for 5 min at RT and slides were mounted with Fluoromount-G solution (Southern Biotech). For the F-actin expression, 1x10 4 primary fibroblasts were seeded on rounded slides into a 24wells plate and incubated o/n 37°C/5% CO 2. The next day were treated with BLM [9µM/1h/37°C /5% CO2], then washed twice in PBS and reincubated in DMEM (10% FBS, glutamine, penicillin and streptomycin) for 4h at 37°C. The slides with adherent fibroblasts treated or untreated were washed in PBS and managed as mentioned above for the EBVB cells up to the staining. Fibroblasts on the slides were firstly incubated with an anti-F-actin antibody (Abcam, ab130935) 1h/RT and detected by a donkey anti-IgM mouse Ax546 conjugated antibody, then stained with AlexaFluor488 mouse anti-γH2AX antibody (BD Pharmangen:560445) 1h/37°C and finally nuclei counterstained with DAPI. Confocal microscopy imaging was performed by Leica TCS-SP8X laser-scanning confocal microscope (Leica Microsystems) using 20x (0.75 NA) objective and 60x (1.42 NA) oil objective for EBVB or 40x for fibroblasts imaging. Single optical sections were acquired with a 1024 × 1024 format and scan speed of 600 Hz. Laser’s power, beam splitters, filter settings, pinhole diameters and scan mode were the same for all examined samples of each staining. Fluorochrome unmixing was performed by acquisition of automated-sequential collection of multi-channel images, in order to reduce spectral crosstalk between channels. MetaMorph software (Molecular Device Inc., Downingtown, PA, USA, Version 7.8) was used to analyse 20x confocal images (at least 14 fields for each sample). After calibration and separation into 8 bits single channel images, the blue channel was thresholded for light objects to identify EBVB nuclei for γH2AX quantification. GraphPad Prism 8.4.3 was used for statistical analysis of γH2AX-integrated intensities (sum of the specific intensities of each pixel in the cell) detected in cells with a diameter of 5-18 µm (14).

*c) Cell viability study:* EBVB cells treated or not with BLM were analyzed for the presence of 7AAD as marker of cell death; the percentage of surviving cells was identified as 7AAD negative cells.

**Statistical Analysis**

Collected data were processed using Prism 6 (GraphPad Software, San Diego, CA). Graphs showed the mean values ± Standard Error Mean (SEM) of data collected by repeated experiments. To compare more than two groups we used the analysis of variance tests: one-way or two way-ANOVA with Bonferroni’s or Dunnet’s or Tukey post-test. Significance levels are as follow: *p<0.05, **p<0.01, ***p<0.001 and ****p<0.0001.

1. Flex E, Martinelli S, Van Dijck A, Ciolfi A, Cecchetti S, Coluzzi E, et al. Aberrant Function of the C-Terminal Tail of HIST1H1E Accelerates Cellular Senescence and Causes Premature Aging. Am J Hum Genet. 2019 Sep 5;105(3):493-508.

2. Bauer CK, Calligari P, Radio FC, Caputo V, Dentici M L, Falah N, et al. Mutations in KCNK4 that affect gating cause a recognizable neurodevelopmental syndrome. Am J Hum Genet. 2018 Oct 4;103(4):621–630.

3. Van der Auwera GA, Carneiro M, Hartl C, Poplin R, Del Angel G, Levy-Moonshine A, et al. From FastQ Data to High-Confidence Variant Calls: The Genome Analysis Toolkit Best Practices Pipeline. Curr Protoc Bioinformatics. 2013; 43:11.10.1-11.10.33.

4. Li H. and Durbin R. Fast and accurate short read alignment with Burrows-Wheeler transform. Bioinformatics. 2009; 25(14): 1754-1760.

5. Cingolani P, Platts A, Wang le L, Coon M, Nguyen T, Wang L, et al. A program for annotating and predicting the effects of single nucleotide polymorphisms, SnpEff: SNPs in the genome of Drosophila melanogaster strain w1118; iso-2; iso-3. Fly. 2012; 6, 80-92.

6. Liu X, Jian X, and Boerwinkle E. dbNSFP v2.0: a database of human nonsynonymous SNVs and their functional predictions and annotations. Hum Mutat. 2013; 34, E2393-2402.

7. Kircher M, Witten DM, Jain P, O'Roak, BJ, Cooper GM, and Shendure J. A general framework for estimating the relative pathogenicity of human genetic variants. Nat Genet. 2014; 46, 310-315.

8. Jagadeesh K, Wenger A, Berger M, Guturu H, Stenson P, Cooper D, et al. M-CAP eliminates a majority of variants with uncertain significance in clinical exomes at high sensitivity. Nat Genet. 2016; 48(12):1581-1586.

9. Li Q and Wang K. InterVar: Clinical Interpretation of Genetic Variants by the 2015 ACMG-AMP Guidelines. Am J Hum Genet. 2017 Feb 2;100(2):267-280.

10. Smedley D, Schubach M, Jacobsen JOB, Köhler S, Zemojtel T, Spielmann M, et al. A Whole-Genome Analysis Framework for Effective Identification of Pathogenic Regulatory Variants in Mendelian Disease. The American Journal of Human Genetics. 2016;99;3;595-606

11. Rausch T, Zichner T, Schlattl A, Stuetz AM, Benes V, Korbel JO. DELLY: structural variant discovery by integrated paired-end and split-read analysis. Bioinformatics. 2012 Sep 15;28(18):i333-i339.

12. Geoffroy V, Herenger Y, Kress A, Stoetzel C, Piton A, Dollfus H, Muller J (2018). AnnotSV: An integrated tool for Structural Variations annotation. Bioinformatics. 2018 Apr 14.

13. Zampetti-Bosseler F, Scott D. Cell death chromosome damage and mitotic delay in normal human, ataxia telangiectasia and retinoblastoma fibroblasts after x-irradiation. Int J Radiat Biol Relat Stud Phys Chem Med. 1981 May;39(5):547-58.

14. Heyn RM, Tubergen DG, Althouse NT. Lymphocyte size distribution. Determination in normal children and adults and in patients with immunodeficiency states. Am J Dis Child.1973 Jun;125(6):789-93.
