# Supplementary material for: Radiosensitivity in patients affected by ARPC1B deficiency: a new disease trait?
Source: Front Immunol. 2022 Jul 29;13:919237. doi: 10.3389/fimmu.2022.919237 (PMC9372879; doi:10.3389/fimmu.2022.919237)
Supplement: Supplementary file 10 [file Table_2.docx]

**Table S2. WES statistics and data output.**

| \| WES enrichment kit \| SureSelect Clinical Research Exome v2 (Agilent) \| \| --- \| --- \| \| Sequencing platform \| Illumina NextSeq500 \| \| Target regions coverage >10x \| 94.3% \| \| Target regions coverage >20x \| 90% \| \| Average depth on target \| 102x \| \| Total number of high-quality variants \| 58,219 \| \| Variants with effect on CDS or affecting splice sites1 \| 12,021 \| \| Private, clinically associated and low frequency variants2 \| 487 \| |
| --- | --- | --- | --- | --- | --- | --- | --- | --- | --- | --- | --- | --- | --- | --- | --- | --- |
| 1 High-quality non-synonymous SNV plus indels within coding exons and splice regions (-3/+8 nt). |
| 2 High-quality, rare/private, functionally relevant variants (gnomAD MAF <0.1%; in house database MAF <1%). |

**Table S3. WGS exonic regions variant summary**

| \| Total number of high-quality variants \| 209,796 \| \| --- \| --- \| \| Variants with effect on CDS or affecting splice sites \| 1,8484 \| \| Private, clinically associated, unknown/low frequency, high predicted impact variants2 \| 280 \| \| Putative disease genes (Autosomal Recessive inheritance)3 \| 88 \| \| Putative disease genes (de novo)4 \| 33 \| \| Candidate genes (Autosomal Recessive inheritance) \| 1, ARPC1B \| |
| --- | --- | --- | --- | --- | --- | --- | --- | --- | --- | --- | --- | --- |
| 1 High-quality non-synonymous single nucleotide variants plus indels within coding exons and splice regions (-3/+8 nt). |
| 2 High-quality, private/rare, functionally relevant variants (CADD phred >= 20.0).  3 NID2 (c.1424C>T, p.Thr475Ile; c.576T>G, p.Asp192Glu), ARPC1B (c.212_226delGCACAGACCGCAACG, p.Gly71_Asn75del), GDF5 (c.826G>T, p.Ala276Ser), LOC101927434 (n.478-122_478-2del), MTERF1 (c.832G>A, p.Gly278Arg), NKD2 (c.65A>G, p.Asp22Gly), RSF1 (c.2222G>A, p.Ser741Asn), SLC6A19 (c.1606G>A, p.Val536Met)  4 SCN5A (c.2783T>G, p.Leu928Arg), WARS (c.1012A>C), ZNF880 (c.1241_1242insATCATGA GGTCAGG AGATCG, p.Lys415fs) |

**Table S4. Variants in other ARPC1B and WAS patient**

| \| Pt1-ARPC1B c.622G>T; p.Val208Phe [4, 5] \|  \| \| --- \| --- \| \| Pt2-ARPC1B c.64+1G>C [4, 5] \|  \| \| Pt3-ARPC1B c.1087dup; p.Glu363Glyfs*95 [4,5] \|  \| \| Pt1-WAS c.961C>T; p.Arg321*  Pt2-WAS c.1291-1292dup; p.Ala433Glufs*13 \|  \| \|  \|  \| |
| --- | --- | --- | --- | --- | --- | --- | --- | --- | --- | --- |
